# Journalists are most likely to receive abuse: analysing online abuse of UK public figures across sport, politics, and journalism on Twitter

**DOI:** 10.1140/epjds/s13688-025-00556-8

**Published:** 2025-05-23

**Authors:** Liam Burke-Moore, Angus R. Williams, Jonathan Bright

**Affiliations:** Public Policy, The Alan Turing Institute, London, NW1 2DB United Kingdom

**Keywords:** Social Media Analysis, Natural Language Processing, Online Abuse, Twitter

## Abstract

Engaging with online social media platforms is an important part of life as a public figure in modern society, enabling connection with broad audiences and providing a platform for spreading ideas. However, public figures are often disproportionate recipients of hate and abuse on these platforms, degrading public discourse. While significant research on abuse received by groups such as politicians and journalists exists, little has been done to understand the differences in the dynamics of abuse across different groups of public figures, systematically and at scale. To address this, we present analysis of a novel dataset of 45.5M tweets targeted at 4602 UK public figures across 3 domains (members of parliament, footballers, journalists), labelled using fine-tuned transformer-based language models. We find that MPs receive more abuse in absolute terms, but that journalists are most likely to receive abuse after controlling for other factors. We show that abuse is unevenly distributed in all groups, with a small number of individuals receiving the majority of abuse, and that for some groups, abuse is more temporally uneven, being driven by specific events, particularly for footballers. We also find that a more prominent online presence and being male are indicative of higher levels of abuse across all 3 domains.

## Introduction

It is by now commonplace for public figures (well known individuals such as celebrities, sports stars, journalists, politicians) to maintain an active presence on social media platforms such as Instagram, Facebook and X (Twitter). Such platforms allow them to build up a personal ‘brand’ and connection with an audience through the generation of a kind of informal, parasocial intimacy [1]; a brand that can then be leveraged for a wide variety of different professional purposes, from securing sponsorship deals [2] to influencing opinions [3] or distributing ideas [4]. Many researchers have highlighted the potential positive consequences of the fact that access to public status is now available through social media channels, in particular by allowing voices into the public sphere who previously would have remained marginalised [5].

However, whilst these positive consequences are important, much of the research on high profile figures on social media has also focused on a negative aspect: the frequency with which these figures receive hateful and abusive messages, facilitated through the peer-to-peer nature of social media. Such messages can be personally distressing for the individuals involved [6, 7], and may lead them to limiting their online presence in order to avoid receiving such messages [8], which in turn will degrade the quality of public discourse and limit the ability for the public to engage with, for example, elected officials. High levels of abuse can also cause more widespread consequences for those witnessing the abuse, who may conclude that public debate is a hostile, angry environment that they should stay away from [9].

Though research on abuse of public figures is widespread, it is also largely siloed, with individual efforts looking at (for example) abuse levels towards politicians, or journalists, or a certain type of celebrity. As methodology can differ across these individual studies, direct comparisons can be complex. We therefore know little about the extent to which dynamics of abuse are similar across different domains, and across demographics within domains. We attempt to advance the debate about abuse towards online public figures by providing a measurement of the extent of abuse faced by three key United Kingdom (UK) based groups (members of parliament, journalists and footballers), and a comparison of the dynamics of abuse between them.

In this paper, we present a cross-group analysis of a novel dataset of 45.5M tweets targeted at 4602 UK public figures across 3 domains, collected between 2021 and 2023, applying our previous work fine-tuning pre-trained transformer models to classify abusive tweets. We structure our enquiry in terms of three key questions:

*Distribution of abuse*: do all domains experience similar levels of abuse, and is this abuse distributed amongst people within the domain in similar ways?

*Temporal patterns*: is abuse a generally stable feature of domains, or does it fluctuate and respond to events?

*Factors linked to abuse*: how does abuse vary with the activity of public figures? After accounting for other potential factors linked to abuse, to what extent is abuse an *intrinsic* feature of a domain – that is to say, to what extent is abuse directly associated with membership of specific demographic groups or domains, rather than driven by other factors such as popularity or online activity? Finally, to what extent is abuse directed at politicians related to their political alignment?

We find that MPs as a group receive more abuse than footballers or journalists. However, through statistical modelling we show that abuse appears to be a greater intrinsic feature of being a journalist. We show that politicians on the left of the political spectrum receive less abuse than those on the right. Lastly, we find that a more prominent online presence and being male are factors predictive of higher levels of abuse, and that abuse is unevenly distributed both individually and temporally across all groups.

## Related work

This paper focuses on the issue of abuse towards public figures on social media. We define abuse as content that threatens, insults, derogates, mocks or belittles an individual or their identity [10]. This is a broad reaching definition that includes but is not limited to more severe forms of abuse that may constitute ‘hate speech’ (hate focused on protected characteristics [11]), also accounting for generic toxicity.

Whilst abuse has been a constant feature of public life, a wide variety of research has found that it appears to be especially prevalent in online discussions. Some of the earliest work on the internet remarked on the apparent prevalence of ‘flaming’ [12], with research continuing to this day about aggressive and uncivil online comments in forums and discussion sections of websites [13, 14]. A considerable further body of research has tried to explain why online environments seem to be so much more hostile than offline ones [15], highlighting factors such as anonymity [16], reduced empathy, and group dynamics where witnessing (and receiving) abuse makes one more likely to create it [17].

Early research on online abuse regarded it largely as an interpersonal phenomenon, exchanged amongst nascent internet communities that were at the time a niche pursuit [18]. However as the internet itself grew into the major means of online societal communication, a further focus developed in terms of the fact that ‘public figures’ also started to become targets. In this paper, we use the term ‘public figures’ to define those whose profession compels them to seek recognition amongst the public and who therefore become known to a potentially wide section of the community. They communicate with an audience of individuals who are unknown to them. Public figures include celebrities, sports stars and famous politicians who might be known to an audience of millions. However they may also include local journalists or members of parliament who might have much lower name recognition but nevertheless have a public face. As Xu et al. [19] show, public figures now exist on all scales, from ‘traditional’ celebrities known to millions to ‘micro-celebrities’. Such figures enter into ‘parasocial’ relationships with members of the wider public: a type of relationship which feels intimate and personal on some level to audience members despite the fact that the public figure themselves is unlikely to have met all of (or even a small fraction of) the audience members with whom they have such a relationship [20].

While public figures have always been a fact of social life, the way they work has changed dramatically with the rise of the internet, and especially social media. These platforms, which provide the possibility of such figures communicating in a relatively direct way with their audiences (circumventing to an extent the filtering mechanisms of the press), have revolutionised what it means to be a public figure, giving rise to a wide variety of new ways of forming parasocial relationships [21, 22]. A presence on at least some social media platforms is by now arguably a requirement of many public facing professions (such as politics and journalism), or at least a highly important way of advancing professional life or monetising status through (for example) endorsements [23].

While access to these platforms arguably represents a boon in many senses, the levels of abuse public figures receive on them is also a source of increasing concern. In this study, we address three different categories of public figure in particular. We look at professional sports stars (in particular football players), journalists, and politicians. This selection does not, of course, address all potential types of public figures (for example, musicians and actors are obvious absences from the list). However, it does offer important variety, with three very different professions with different audiences to communicate with, who have a large presence on social media, and are well-noted for receiving large volumes of abuse. They also are groups that are clearly delineated, with members having specific status (e.g by football leagues, elected position, job title), and in the case of Footballers and MPs, are of a finite number.

Each of these categories has attracted a considerable amount of research on levels of abuse, which we will review in turn below. What is missing, which we provide here, are studies that address multiple categories in the same framework, and thus provide a more general view on the dynamics of abuse.

### Football

Abuse towards professional athletes (and other related professionals such as referees) has long been part of the sporting industry [24], and in the past has been associated with multiple campaigns launched by the industry itself to attempt to stamp it out [25]. While there was some perceptions that these campaigns had been partially successful, the rise of social media as a forum for the self-presentation of footballers for many brought about a kind of regression with abuse once again rife, also closely associated with the issue of racism [26]. Empirical work has consistently documented relatively high levels of abuse towards footballers [27, 28]. However the vast majority of quantitative studies have, to our knowledge, been directed towards male sports stars, despite obvious press attention to abuse towards female athletes as well [29]. Concerns about abuse (especially racist abuse) being directed towards footballers are based of course on the mental health and wellbeing of the players themselves, with players having even contemplated suicide when being on the receiving end of it [29] and family members also feeling an incredible amount of strain [30]. Furthermore, due to the highly mediatized nature of the phenomenon, there are concerns that witnessing of online abuse may serve to normalise it in wider society.

### Politics

Volumes of abuse towards professional politicians have also been a subject of considerable research interest. The world of professional politics is of course a combative one, with threat and harassment unfortunately a part of life for professional politicians of all types [31, 32]. The online arena seems to be an extension of this trend, with a wide variety of work documenting the high levels of abuse and vitriol directed towards elected officials [33, 34], and some also arguing that the problem is increasing over time [35, 36].

One of the key debates in this area is whether male and female politicians experience different volumes and types of abuse, with some studies not identifying gender differences [33, 37], whilst others have problematised this type of finding [38–42] or shown mixed results [43]. The recent resignations of high profile female politicians, citing patterns of abuse received, seem highly significant in this regard [44, 45]. A similar but smaller body of literature has also sought to highlight religious and racial differences [46]. Another key debate is the reasons for abuse [37], with some arguing that periodic news attention to different topics is a key driver and others pointing to differences in the profile of the individuals in question [35]. These issues are critical not only in terms of effects on politicians themselves and wider public discourse, but also in terms of concerns about impacting on the representativeness of democracy as a whole.

### Journalism

The impact of online abuse on the journalistic profession has also attracted a considerable amount of scrutiny (including noting the clear crossovers with the previous two domains in terms of journalists covering both sports and politics). Findings are in a sense similar to the other two domains: first, a wide variety of scholarship has claimed the problem is serious and widespread [47–49], as well as being connected to real world acts of violence, a subject of particular concern as many journalists lack the security protections provided to politicians (though this is not to say that politicians do not also frequently experience violent attacks). Diverging patterns of abuse between men and women have also been a frequent area of study [50–52]: though unlike in the political arena, greater levels of abuse directed towards women has been a clear and consistent finding [47, 53].

Personal visibility (as opposed to newsroom visibility) is suggested as another factor of the abuse of journalists [53], and many studies have also linked it to broad societal factors such as the rise of populism [48, 49]. Temporal factors have also been considered, with work describing online abuse towards journalists as both a chronic problem and one that is also likely to be boosted by individual events [54]. Some of the feared consequences of abuse for journalists are also somewhat similar to the political domain: that the distress and psychological burden created by abuse patterns will drive people out of the public domain [47, 55]. However, authors have also noted that this abuse may create a more general perception in the eyes of journalists themselves that news audiences are irrational and low quality [53].

### Other public figures

While we do not directly address them in our empirical work, it is also worth briefly reviewing here some other types of public figures that have faced abuse online, to highlight the connections of our work to other domains. Film and television stars are an obvious category who are frequent targets of online abuse, often linked to perceptions of their role in theatrical releases. Some reaction is linked to negative perceptions of performances. However, like some of the domains above, researchers have highlighted the relationship between abuse and the gender and ethnicity of actors playing roles. For example, increasing diversity in long standing franchises such as the Marvel comics series, Ghostbusters and Star Wars has resulted in campaigns of hate directed towards both stars of the movies and the companies that produced them [56], [57], [58]. Impact on the indviduals concerned can be considerable, for example actor Ahmed Best (who played a character in one of the Star Wars movies) stated that “fan backlash was so fierce that he considered taking his own life” [57].

Beyond film stars, there are many other types of public figure who experience similar patterns. Social media influencers face abuse: indeed, the experience is particularly widespread, with a recent survey finding that over 70% of influencers had faced some kind of toxicity or negative reaction online [59]. Participants in reality TV shows have also been highlighted as targets, with the hate they receive mixed up with the overall experience of watching the show and ‘judging’ its participants [60]. Researchers themselves can also become targets, and will often modify their online behaviour considerably in response [61]. In short, online hate and abuse towards public figures is a widespread phenomena.

One of the most significant and yet under-explored things emerging from all of this work is that, despite the great differences in the profession and style of work these different public figures are employed in, many similar patterns and claims about online abuse have emerged. However, what the field as yet lacks is comparative work looking at different professions to tie these observations together (one notable exception is [62], though this looks only at female journalists and politicians). We hence lack knowledge about what features of abuse are unique to a given professional context and what are more general features of online public life as a whole.

In this article, we seek to remedy this deficit, by measuring levels of abuse across our three different domains of interest. Our aim is to describe the dynamics of online hate and abuse towards public figures in a way that is not entirely dependent on the domain or field of study.

## Methods

### Platform selection

In this paper we make use of data collected from the social media platform Twitter/ X (we refer to “Twitter” exclusively given data collection took place primarily before the rebrand to “X”). As a platform focused on broadcasting messages, with no requirement for reciprocal following before messages are exchanged, Twitter has long been a forum where public figures have maintained an active presence and broadcast messages to an audience. It hence represents an ideal choice of venue for our study. It is worth noting that other platforms are also being used by public figures, with the footballers in our study also highly present on Instagram, for example. However, there is no other platform that is widely used by all the groups in our study.

### Target group selection

We select 3 domains of public figures to study: Premier League football players, UK members of parliament, and journalists. As discussed in Sect. 2, these 3 groups are clearly identified individuals, and, for footballers and MPs, are of a finite number, enabling more precise data collection. We delineate public figures into male and female groups (this study is limited to binary gender, given the low prevalence of individuals identifying as non-binary or other genders within these groups). This gives us 6 individual groups across 3 domains and 2 genders.

We source lists of public figures from official sources where available, and filter lists down to include only those with an official Twitter account. Full details are visible in Appendix A. The final, total number of individual public figures, present on Twitter, across all domains and demographics, included in this study are visible in Table 1. Immediately apparent is the minority of female public figures across all domains. This is mirrored by follower accounts, where the average man individual public figure has a higher follower count than the average woman, and the majority of the top 10 most followed individuals in each domain-demographic pair are men. This could be seen as a feature of the domains themselves (and society in general): while the popularity of women’s football grows, men’s football receives more widespread engagement [63], the balance of female MPs was below 20% until 2006 [64], and journalism has been shown to be an industry dominated by men [65].

**Table 1:** Counts of public figure Twitter accounts

Domain	Gender		Total
	*Female*	*Male*	
Footballers	204	807	1011
Members of Parliament	207	384	591
Journalists	1210	1790	3000

### Data collection

Central to Twitter activity are primarily text-based posts called “tweets”. Tweets can be replied to, creating chains or “threads”, or can be reposted as “Quote tweets” or “Retweets” of other tweets. We are interested in the most direct form of communication targeted at public figures, and as such we only consider what we term *“audience contact”* (AC) tweets: direct replies to a tweet from a public figure account, or top-level tweets (that aren’t replies to other tweets) containing a mention of a public figure account. We present aggregate statistics based on these tweets and the labels assigned.

We use the Twitter application programming interface (API) Filtered Stream endpoint and Full Archive Search endpoint (provided by the Twitter Academic API, no longer available) to collect all tweets that either contain a mention of a public figure account (including direct and indirect replies) or are quote tweets or retweets of tweets created by a public figure account. Data collection endpoint usage and time windows differed across domains and demographics, as outlined in Table 2, due to the staggered nature of data collection for this project. All data collection ended on the 14th of March 2023, when API access was suspended. We filter the tweets collected to retain only tweets matching the *audience contact* conditions, that are written in English, and contain text content aside from mentions and URLs. On collection, we extracted lists of public figure accounts mentioned within the tweet text, and created a clean version of the tweet text, replacing mentions of users with domain-specific tokens, and URLs with a URL token. The remaining “valid” *audience contact* tweets (visible in Table 2) for each domain-demographic pair are used for the modelling and analysis presented in this paper.

**Table 2:** Table of data collection windows and Tweet counts per domain- demographic group. “Group” column denotes domain-demographic pair in the form “DOMAIN; GENDER”. “Tweets” represents the number of valid audience contact tweets. “Median Followers” represents the median average number of followers within the group

Group	Start date	End date	Total days	Tweets	Median followers
FB;M	12/08/2021	14/03/2023	579	7,398,876	37,184
FB;W	12/08/2021	14/03/2023	579	303,403	6442
MP;M	13/01/2022	14/03/2023	425	18,741,751	15,845
MP;W	13/01/2022	14/03/2023	425	9,404,846	21,266
JN;M	01/08/2022	31/01/2023	183	7,300,005	9950
JN;W	01/08/2022	31/01/2023	183	2,343,299	8309

We additionally collect all tweets authored by public figure accounts, again using the Twitter API Filtered Stream endpoint and Full Archive Search endpoint, in order to enable analysis around the activity of public figures and the relationship with abuse. We retain all of these tweets, regardless of language and content, and we do not label these tweets as abusive / not abusive.

### Abuse classification

We fine-tune pre-trained transformer-based language models for binary abuse classification for each public figure target group, using the models and data presented in Vidgen et al. [27] and Williams et al. [10].

All tweets are annotated with one of four labels: “abusive”, “critical”, “neutral”, or “positive”. Definitions of each class, and guidelines for annotators, are visible in Fig. 8. Here we define abuse as broad-reaching, including but not limited to hate speech, pertaining to any content that threatens, insults, derogates, mocks or belittles an individual or their identity [10]. We collapse multi-class labels to binary labels (abuse as positive, any other class as negative) for the models in this study. At least 7000 tweets are annotated for each group: 1000 for the validation split, 3000 for the test split, and 3000 for the training split (more in the case of male footballers).

Initial rounds of annotation (the male and female footballers datasets) were done by crowdworkers, but, due to high levels of disagreement between crowdworkers, and therefore more expert annotation required, a small group of high-quality annotators was used to label the remaining datasets (MPs, Journalists).

For *male footballers*, we use a version of deBERTa-v3 [66] fine-tuned on 9500 tweets targeted at male footballers. This model is trained using an active learning process, starting with a sample of 3000 tweets, and using diversity and uncertainty sampling to select 2000 additional training entries to annotate over 3 rounds, plus one round of 500 adversarial entries, as outlined in Vidgen et al. [27]. Tweets were annotated by 3375 crowdworkers. This model outperformed (F1 score on the male footballers test split) a model trained on the base 3000 male footballers training dataset, and an ensemble of two models trained on male footballers and female footballers data.

For *female footballers*, we use an ensemble of two fine-tuned versions of deBERTa-v3 [66], one on tweets targeted at male footballers, the other on tweets targeted at female footballers. Both models were fine-tuned on 3000 tweets, as outlined in Williams et al. [10]. Tweets were annotated by 3513 crowdworkers. Output probabilities from the two models are averaged during inference to make classifications. This ensemble outperformed (F1 score on the female footballers test split) the model trained on the base 3000 female footballers training dataset.

For *MPs*, we use an ensemble of two fine-tuned versions of deBERTa-v3 [66], one on tweets targeted at male MPs, the other on tweets targeted at female MPs. Both models were fine-tuned on 3000 tweets, as outlined in Williams et al. [10]. Tweets were annotated by 23 high quality annotators. Output probabilities from the two models are averaged during inference to make classifications. This ensemble outperformed models trained on the base training datasets for both male and female MPs.

For *journalists*, we use a version of deBERTa-v3 [66] fine-tuned on 3000 tweets targeted at journalists, following the same processes outlined in Williams et al. [10]. Tweets were annotated by the same 23 high quality annotators as the MP datasets.

Model evaluation results are visible in Table 3. The models displayed were chosen for superior performance over e.g. individual models used in ensembles, with respect to time and resource limitations. Performance of all candidate models is visible in Table 10.

**Table 3:** Evaluation of models used for abuse classification. “Group” column denotes domain-demographic pair in the form “DOMAIN;GENDER”

Group	Model description	Train size	F1 score	Precision	Recall
FB;M	deBERTa-v3 model fine-tuned on male footballer data through active learning [27]	9.5K	0.65	0.65	0.64
FB;W	Ensemble of 2 deBERTa-v3 models, fine-tuned on male footballer data and female footballer data [10]	2*3K	0.62	0.79	0.51
MP;M	Ensemble of 2 deBERTa-v3 models, fine-tuned on male MP data and female MP data [10]	2*3K	0.68	0.71	0.65
MP;W			0.61	0.67	0.56
JN;M	deBERTa-v3 model fine-tuned on journalist data	3K	0.62	0.61	0.64
JN;W			0.66	0.66	0.66

This set of models forms part of our attempt to provide a more consistent examination of the abuse of public figures. As a result, performance recorded is not necessarily state of the art within the literature. Further details on error analysis are visible in [10]. Our analysis primarily examines aggregate patterns of abuse rather than making decisions about individual tweets, which reduces the impact of individual misclassifications.

## Results

We structure our analysis in terms of our three research questions.

### Distribution of abuse

We begin our analysis by assessing whether all domains experience similar levels of abuse, and look at whether this abuse is distributed amongst individual public figures within a domain in similar ways.

We present total tweet counts in Table 4 and weekly average tweet counts in Table 5 (total counts are presented for completeness, weekly averages are used for analysis due to variable time windows between domains). We see that MPs have the highest weekly average rate of abuse, with 11.2% of tweets received by male MPs being classified as abusive, and 9.1% for female MPs. We also see that male footballers receive a higher average proportion of abuse containing identity-based slurs than any other group at 11%. 32 journalists received no Tweets during the data collection window.

**Table 4:** Total metrics per domains and demographic group. “Group” column denotes domain-demographic pair in the form “DOMAIN;GENDER”

Group	Tweets authored	Total tweets received	Abusive tweets received	Abuse proportion	Identity-abuse proportion
FB;M	31,776	7,398,876	186,085	2.5%	11.3%
FB;W	13,405	303,403	2599	0.9%	5.8%
MP;M	73,535	18,741,751	2,131,022	11.4%	5.9%
MP;W	46,190	9,404,846	884,493	9.4%	4.9%
JN;M	464,132	7,300,005	535,631	7.3%	5.9%
JN;W	149,879	2,343,299	159,516	6.8%	5.0%

**Table 5:** Weekly average metrics per domain and demographic group. “Group” column denotes domain-demographic pair in the form “DOMAIN;GENDER”

Group	Tweets authored	Total tweets received	Abusive tweets received	Abuse proportion	Identity-abuse proportion
FB;M	942	88,082	2215	2.5%	11.0%
FB;W	201	3528	30	0.6%	5.6%
MP;M	1559	302,286	34,371	11.2%	6.0%
MP;W	931	151,691	14,266	9.1%	5.0%
JN;M	18,360	270,371	19,838	7.2%	5.9%
JN;W	6034	86,789	5908	6.8%	5.0%

**Figure 1:**
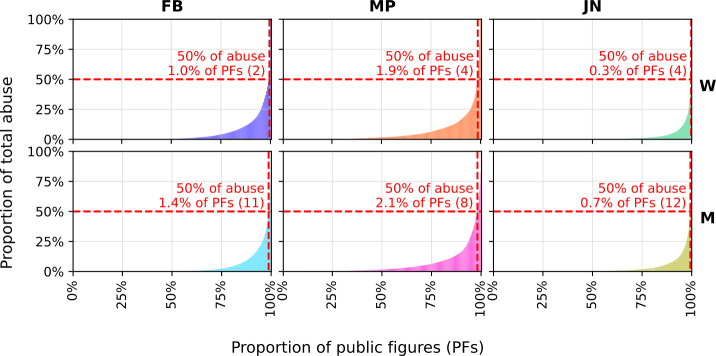
Cumulative distributions of abuse by individual public figure. One column per domain, one row per gender. Proportion of the required top N% of public figures to reach 50% of all abuse is annotated

We present cumulative distributions of total abuse counts by individual public figures in Fig. 1. We see that, across all domains and demographics, a small number of individuals receive a large proportion of the total abuse. For example, 50% of abuse targeted at male MPs is directed towards just 2.1% of all of those individuals. This observation holds for other domains and demographics, although differences can be partly explained by the differing number of public figures in each group.

**Figure 2:**
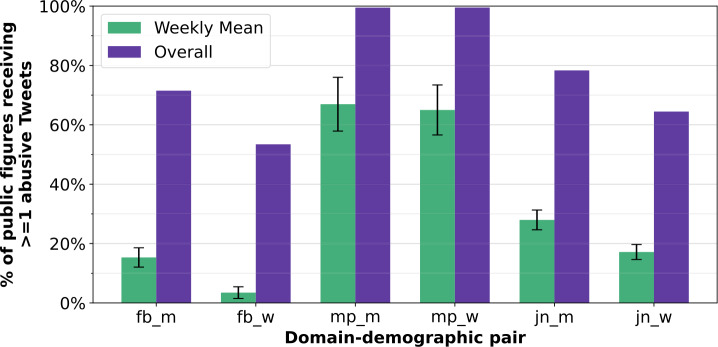
Weekly average and overall proportions of public figures receiving at least one abusive tweet. Shows standard deviation, and overall proportion of public figures who received at least one abusive tweet during all data collection

The proportion of public figures that received any abuse across the entire data collection window is visible in Fig. 2, showing that almost all MPs (99.5% for men and women) received any abuse, and over 50% of footballers and journalists. The different lengths of the data collection windows does affect this, meaning we might see higher levels of coverage for journalists if the data collection window was more similar to that of MPs and footballers, but the fact that relatively few footballers (71.5% of men, 53.4% of women, lowest across both demographics) received any abuse, despite the data collection window being the largest of the 3 domains, does emphasise that fewer footballers receive any abuse at all than the other domains.

One might assume that the most abused public figures are also the most popular public figures. We use the Spearman rank correlation between the quantity of abuse a public figure receives and the number of followers they have, visible in Table 6.

**Table 6:** Spearman rank correlation coefficients between abuse received and number of followers. Coefficients are presented for all public figures within a group, and for the top 50 in terms of abuse received

Domain	Demographic	Rank Correlation	
		All	Top 50
Footballers	Men	0.83	0.39
	Women	0.71	0.52
MPs	Men	0.53	0.45
	Women	0.49	0.60
Journalists	Men	0.57	0.23
	Women	0.48	0.51

We see a mild to strong rank correlation between abuse and followers when considering the total population of each group of public figures, highest for footballers (0.83 for men, 0.71 for women). However, when we limit the analysis to only the top 50 most abused public figures from each group, this changes - there is still a positive rank correlation for all groups, but the correlation is significantly weaker for footballers and male journalists. This indicates that some of the most abused individuals within these groups have lower follower counts. This points to more circumstantial abuse centered around specific events, and more work is needed to understand this phenomenon.

### Temporal patterns

It is well known that abuse of public figures is not a stable phenomenon temporally [27], with abuse rising and falling in relation to real world events. Here, we explore the extent to which abuse levels fluctuate over time, and how the dynamics of temporal fluctuation differ by domain and demographic.

**Figure 3:**
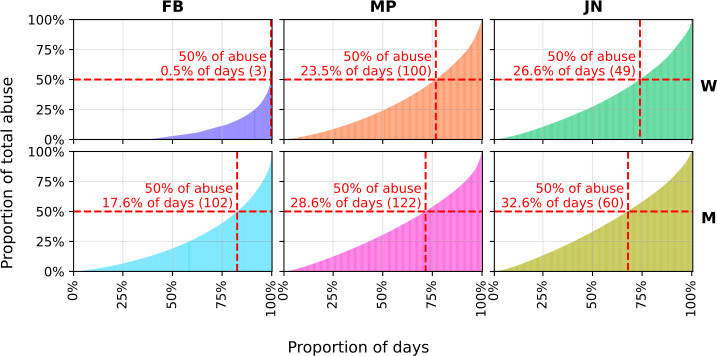
Cumulative distributions of abuse by day. One column per domain, one row per gender. Proportion of the required top N% of days to reach 50% of all abuse is annotated

We present cumulative distributions of total abuse counts by day in Fig. 3. We see that abuse tends to be unevenly distributed over time for all groups. 50% of abuse targeted at footballers takes place over 9.1% of days (on average across both men and women). Abuse towards MPs and journalists is more evenly distributed, with 50% of abuse taking place over 26.1% and 29.6% of days respectively on average. Within each domain, abuse of female public figures tends to be more uneven than for their male counterparts. Female footballers receive 50% of their abuse over just 0.5% of days compared to 17.6% of days for male footballers. This observation holds to a lesser extent for MPs and journalists, where the difference between female and male public figures is 5.1% and 6.0% respectively.

**Figure 4:**
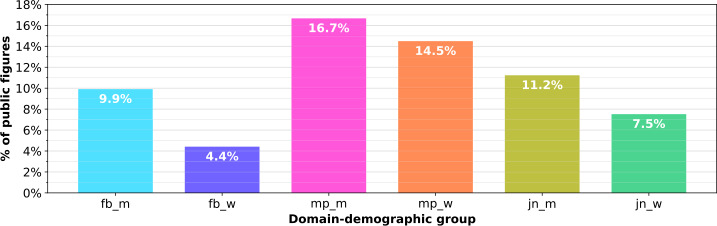
Percentages of public figures who received at least a third of their total abuse in one day. Only includes public figures who received at least 10 abusive tweets during data collection

Investigating temporal fluctuation at an individual level, we present percentages of public figures who receive at least ${\frac{1}{3}}$ of their total abuse in a single day in Fig. 4. This shows the presence of public figures in all groups who receive a significant proportion of the abuse they receive throughout the whole study in a single day. More MPs than any other group receive over ${\frac{1}{3}}$ of their abuse in a single day (15.6% on average), followed by journalists (9.4% on average), and then footballers (7.2% on average). Across all domains, more men receive a significant proportion of their abuse in a single day than women.

**Figure 5:**
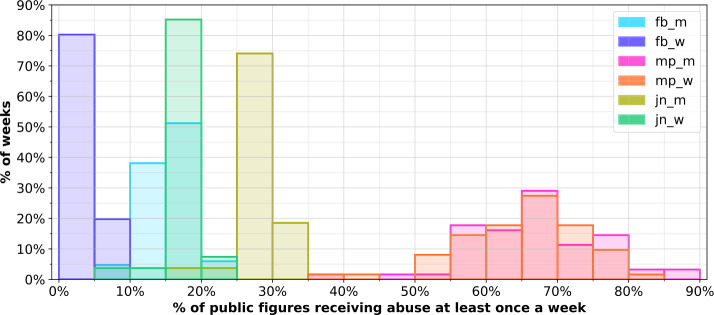
Histograms of percentages of public figures receiving at least one abusive tweet in a week

Finally, in Fig. 5 we plot histograms of weekly percentages of public figures receiving any abuse within a given week. We see that the majority of MPs receive abuse on a weekly basis, with over 50% of MPs receiving at least one abusive tweet in all but 2 (3.2%) weeks during data collection. No other group receives such regular and widespread abuse - at no point do over 50% of footballers or journalists in our dataset receive at least one abusive tweet in a single week, with the average being 15.3% for male footballers, 3.47% for female footballers, 28.0% for male journalists, and 17.2% for female journalists, compared to 67.0% for male MPs and 65.0% for female MPs.

Taken together, we make several observations about these results. Firstly, MPs as a group receive the most regular and widespread abuse, but also individually see many concentrated periods of abuse. This suggests that whilst abuse may be a stable feature of being an MP, there is also a large element of this abuse which is more unstable and sporadic. These abusive tweets could be in relation to specific events, for example, in response to a controversial tweet or comment made by an MP.

Footballers, on the other hand, receive the least regular and widespread abuse as a group. In most weeks a small proportion of players receive any abuse, which tends to be less evenly distributed over time. This suggests that abuse towards footballers is more sporadic and event driven. However, compared to MPs, a smaller minority of players receive a significant proportion of their abuse in a single day. It may be the case that whilst footballers receive less regular abuse than MPs *as a group*, many individual footballers receive more regular abuse spread out over specific days, such as match days. One can imagine this as a series of regular peaks in abuse between troughs of low abuse levels, resulting in an uneven distribution but lacking individual peaks that account for significant proportions of abuse.

The temporal nature of abuse towards journalists is somewhere between that of MPs and footballers. In most weeks a reasonable proportion of journalists receive some abuse, which is more evenly distributed over time than for MPs or footballers. The proportion of journalists who receive at least a third of their abuse in a single day (9.35% on average across men and women) is less than MPs (15.6% on average) but greater than footballers (7.15% on average).

On an individual level, less women receive over ${\frac{1}{3}}$ of their abuse in a single day than men (across all domains), suggesting a more even distribution for abuse of female public figures. However, on a group level, abuse towards women is in fact less evenly distributed over time than for men. This requires more analysis to understand, but may be due to the presence of events that see female public figures abused as a group, to a greater extent than for male public figures.

Investigating the most prevalent peaks in abuse noted in Fig. 9 for each group of public figures, we see some differences across groups, with the majority of peaks relating to real world events, and some in response to social media comments made by public figures.

The peaks for footballers relate to transfer activity (e.g. fans upset the player is leaving them for another team), leaked footage of a footballer committing animal abuse, and backlash to comments made on social media by a female footballer.

The 3 peaks for male and female MPs align to the same 3 (separate) weeks, where significant revelations around misbehaviour and the resulting political fallout occurred, followed by changes in leadership, and the potential return of controversial figures to cabinet roles, wherein some MPs also drew high levels of abuse as a result of voicing their support of (or opposition to) these individuals. Some of the peaks for abuse of journalists align to these peaks for MPs, and relate to journalists discussing these events and drawing abuse due to their opinionated coverage of the news. Other peaks for journalists occurred due to controversial posts on social media that caused significant backlash.

### Factors linked to abuse

We tackle our third and final research question regarding which factors are linked to abuse. We firstly examine the relationship between the activity of public figures and the abuse they receive, and then attempt to quantify how intrinsic abuse is to a domain or gender through statistical modelling. We finally examine the relationship between political alignment the levels of abuse directed towards politicians.

#### Public figure activity

We count the number of “active statuses” written by a public figure (the number of statuses posted by a public figure account that receive at least one reply) as a measure of their activity.

Looking at weekly average activity in Table 5, journalists appear as the most active group by a significant factor, with MPs more active than footballers on average by a smaller margin. This holds when accounting for the number of public figures studied, given the larger number of journalists - the average journalist writes 7.2 (9.7 for men, 4.6 for women) tweets per week, compared to 3.3 (3.1 for men, 3.6 for women) for the average MP and 0.6 (0.8 for men, 0.5 for women) for the average footballer.

**Figure 6:**
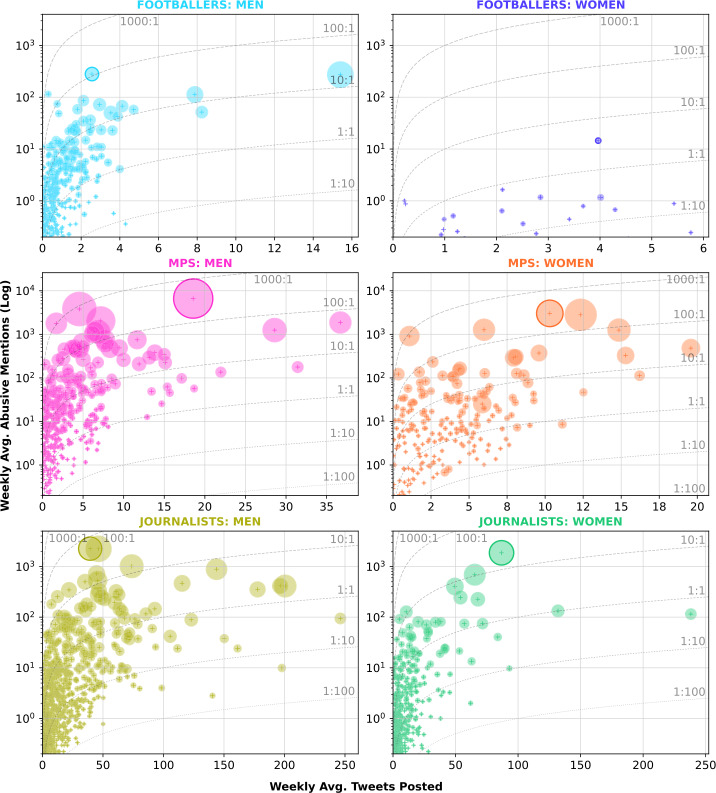
Public figure activity against abuse received. Dotted lines represent ratios of abusive mentions to Tweets posted by a public figure. Size of markers represents total tweets received by the public figure. The most abused public figure in each domain is marked with a solid outline

Figure 6 visualises the relationship between activity and abuse for public figures across different domains. In terms of ratios of abuse to activity, only considering public figures receiving at least 1 abusive tweet per week on average, we see 28 male and 5 female MPs receiving at least 100 abusive tweets for every tweet they write per week, compared to 9 and 4 journalists, and 3 and 0 footballers. The average ratio is also higher for MPs, at 32.1 abusive tweets per status for men and 22.7 for women, versus 17.2 and 1.9 for footballers and 1.4 and 1.6 for journalists.

There appears to be some positive relationship between activity and abuse. Correlation coefficients between abuse and activity are mild but positive, with the average Pearson correlation coefficients at 0.32, strongest for male footballers (0.64). However, across all groups, the most active individual is never the most abused individual, but does still consistently rank in the top 8% of public figures. This suggests that, while some level of activity may be a pre-requisite to receiving higher levels of abuse, it doesn’t necessarily identify the most abused individuals.

#### Intrinsic nature of abuse

In Sect. 4.1 and Sect. 4.2 we use *absolute* levels of abuse to compare across domains, demographics, and time. This is important as it affords us a better understanding of how public figures actually experience abuse online. However, this approach does not allow us to assess the extent to which abuse is *intrinsic* to a domain or demographic – that is, whether abuse is a direct result of belonging to a particular domain or demographic, or whether it can be explained by other factors irrespective of domain or demographic (such as the prominence or activity of a public figure).

To examine whether abuse is directly associated with domain or demographic membership, we fit a series of count models. Each observation in these models represents a single public figure and the dependent variable is the number of abusive tweets received by that individual. We first fit separate models to each domain to examine gender differences within these groups. We refer to these models as Model 1 (Journalists), Model 2 (MPs), and Model 3 (Footballers). In each of these models, our independent variable of interest is *gender*, with female gender serving as the reference category.

We then fit an additional model (Model 4) to assess differences between domains. In this model we are interested in the independent variable *domain*, with the footballer domain serving as the reference category. Model 4 also includes an exposure offset term to account for the differing time periods in which data were collected for each domain. This was set to the log of the total number of weeks of data collection for each domain. In all models we control for the following variables:

*Count total*: the total number of audience contact tweets received by the public figure.

*Count followers*: the number of followers each public figure has.

*Count replied to*: the number of tweets written by the public figure that received at least one reply.

To assist with model convergence, and to aid interpretation of results, these variables were incremented by 1 and log_2_ transformed. We summarise the four models and their variables in Table 7.

**Table 7:** Summary of models

Model	Domain	Dependent variable	Independent variables
1	Journalists	No. abusive tweets	Count total, Count followers, Count replied to, Gender
2	MPs	No. abusive tweets	Count total, Count followers, Count replied to, Gender
3	Footballers	No. abusive tweets	Count total, Count followers, Count replied to, Gender
4	All domains	No. abusive tweets	Count total, Count followers, Count replied to, Gender, Domain

For each model, we use a likelihood ratio test (LRT) to determine whether a Poisson or a negative binomial regression is most appropriate. As the Poisson model is nested within the negative binomial model, the LRT is a suitable test to compare the fit of these models. All tests indicated the negative binomial provided a significantly better fit. As the negative binomial model estimates a dispersion parameter, which is held constant in the Poisson model, this suggests our data is over-dispersed. We considered using zero-inflated versions of these models (i.e. zero-inflated Poisson and zero-inflated negative binomial), but the lack of a strong theoretical reason for the existence of excess zeros deemed these inappropriate. Negative binomial models were run using the MASS R package [67], and approximate 95% confidence intervals were obtained by likelihood profiling. We report incident rate ratios (IRRs) by exponentiating the raw model coefficients. For a log_2_ transformed count variable, the resulting IRR represents the multiplicative change in incident rate when that count is doubled.

**Figure 7:**
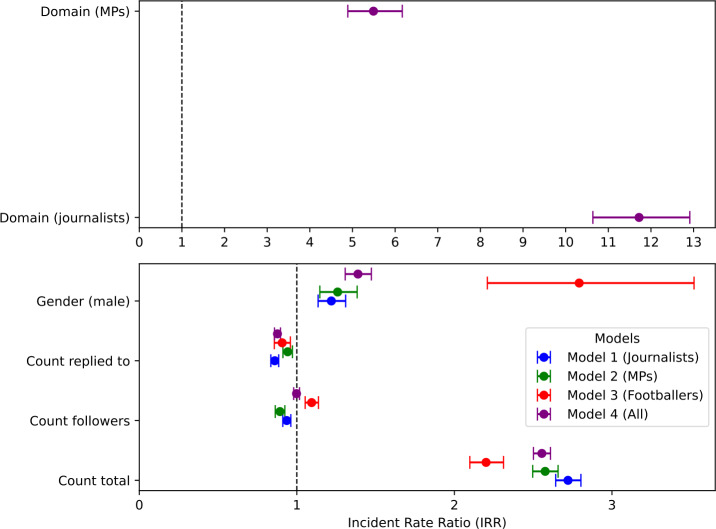
Forest plot of IRRs and confidence intervals

Models were checked for multicolinearity by calculating variance inflation factors (VIFs), with VIFs for all variables in all models no greater than 5. Outliers were also checked for using Cook’s distance (CD). A cutoff of $4 / N$ (where *N* is the number of observations) is typically used to identify potential outliers. Data points with a CD greater than or equal to this cutoff were removed, models refit, and the resulting coefficients checked for changes in direction and significance. Two changes were observed after refitting models. In Model 3, the control variable *Count replied to* was no longer deemed significant, whilst in Model 4 the raw estimate for *Count followers* changed from -0.002 to 0.003 (representing a change in the corresponding IRR from 0.998 to 1.003).^1^ Taken together, these diagnostics suggest no reason to doubt the reported results. We present results for all models in Table 8, and a forest plot of IRRs and associated confidence intervals in Fig. 7.

**Table 8:** Negative binomial model results

Term	IRR	95% conf. interval
*Model 1 (Journalists)*		
Count total	2.721	2.643 - 2.803
Count followers	0.936	0.911 - 0.962
Count replied to	0.860	0.835 - 0.885
Gender (male)	1.219	1.135 - 1.310
*Model 2 (MPs)*		
Count total	2.576	2.497 - 2.658
Count followers	0.893	0.863 - 0.924
Count replied to	0.942	0.912 - 0.972
Gender (male)	1.259	1.146 - 1.383
*Model 3 (Footballers)*		
Count total	2.201	2.098 - 2.312
Count followers	1.094	1.053 - 1.137
Count replied to	0.907	0.857 - 0.959
Gender (male)	2.792	2.209 - 3.521
*Model 4 (All)*		
Count total	2.555	2.502 - 2.610
Count followers	0.998	0.980 - 1.017
Count replied to	0.877	0.858 - 0.896
Gender male	1.388	1.307 - 1.473
Domain (journalists)	11.721	10.638 - 12.911
Domain (MPs)	5.492	4.891 - 6.168

Models 1 – 3 look at how the levels of abuse received by public figures varies by gender. In all three models male public figures experience more abuse than female public figures. On average, and with all other variables held constant, male journalists receive a 22% greater incidence of abusive tweets than their female counterparts, whilst male MPs receive a 26% greater incidence. Male footballers receive an incidence that is almost three times greater than for female footballers. The 95% confidence intervals do not contain 1, and so these estimates can be considered statistically significant.

Model 4 looks at how abuse levels differ between domains. The results show that on average, and with all other variables held constant, journalists receive abuse at a rate which is almost 12 times that for footballers. The rate at which MPs receive abuse is almost 6 times that for footballers. Again, since the confidence intervals do not contain 1, these estimates can be considered statistically significant at the 95% level.

#### Political alignment

Finally, we assess the extent to which abuse directed at politicians is associated with their political alignment. We focus the analysis on two UK political parties: the Labour Party and the Conservative Party. This is for two reasons. Firstly, these two parties currently dominate UK politics, with most MPs belonging to one of the two. Secondly, it is difficult to assign a ‘political alignment’ to several of the smaller parties, whose policies sometimes straddle the political spectrum. Focusing on the two largest parties whose political alignment is much more clearly delineated (with Labour being centre-left to left-wing and Conservatives being centre-right to right-wing) allows us to more readily compare levels of abuse on two sides of the political spectrum.

To do this we adapt Model 2 from Sect. 4.3.2 by including an additional independent variable *Party*, where Conservative Party serves as the reference category. We restrict the analysis to those MPs who are in either the Conservative Party or the Labour Party only. We follow the same procedure as before to obtain IRRs and confidence intervals, which we present in Table 9. The results show that on average, and with all other variables held constant, Labour MPs receive abuse at a rate 36.4% lower compared to Conservative MPs. The 95% confidence interval does not contain 1, and so this estimate can be considered statistically significant at the 95% level. We also note that the IRR for male MPs is slightly reduced from 1.259 in Model 2, although it is still considered statistically significant.

**Table 9:** Negative binomial model results for political alignment analysis

Term	IRR	95% conf. interval
Count total	2.418	2.326 - 2.514
Count followers	0.951	0.914 - 0.991
Count replied to	1.002	0.965 - 1.040
Gender (male)	1.188	1.074 - 1.314
Party (Labour)	0.636	0.557 - 0.726

## Discussion and conclusion

Overall, we find that MPs receive higher absolute levels of abuse at a more constant rate than footballers or journalists, although abuse appears to be a greater intrinsic feature of being a journalist than it is for an MP or footballer. We also find that Conservative MPs receive higher levels of abuse than Labour MPs. Across all domains, the majority of abuse is directed at a very small number of individuals, but the majority of all public figures studied across each group received at 1 least abusive tweet during the data collection window. Abuse levels are more evenly distributed over time, but fluctuate to a much greater degree for footballers than MPs or journalists. We find that abuse levels tend to be higher for public figures who are more active or have more followers, but the most abused individuals are rarely the most active or most followed. Across all domains, an average male public figure receives more abuse than an average female public figure, but also has more followers and receives more tweets in total - controlling for these factors, statistical models still indicate that being a man is predictive of higher abuse levels. We also see that abuse targeted at female public figures fluctuates with time to a greater extent than male public figures, and is therefore likely to be driven by specific events.

### Limitations

In this study we focus on a broad-reaching definition of abuse, and count the number of tweets that meet that definition (as classified by a machine learning model). This does not account for the potential range of severity of abuse, and as such all abuse counts equally towards the figures presented, lacking the nuance that some public figures may receive higher levels of more severe abuse than others. In the same vein, abuse may affect individuals in different ways, and measuring counts of tweets does not encapsulate the impact of abuse on individuals. As such, our results are best interpreted as counts of abusive language, with further work needed to understand how the severity and impact of abuse differs across groups of public figures.

We restrict our analysis of tweets to those in the “audience contact” category in order to increase the chance that any given tweet is directly addressed towards a public figure. However, we recognise that our approach is not perfect and may inadvertently capture tweets not targeting these individuals. For example, we did identify tweets that targeted other public figures, targeted other users within the thread, or even used abusive language to show support for a public figure. Our results, therefore, may reflect levels of hostile discourse surrounding a public figure more generally rather than abusive targeting of that individual in particular. Our results should be interpreted with this in mind.

Our focus is on “audience contact” tweets, however we recognise that abuse doesn’t solely exist within this category on social media - many public figures receive abuse via direct messages, which are not available for collection, and abuse may also take place without mention or reply to the subject of the abuse. As noted earlier, this study is limited to a single platform, and as such conclusions can only be drawn within the scope of Twitter/X.

We delineate public figures into binary gender categories. As noted, we do not include other possible gender identities due to low prevalence within the groups studied. A public figure from a minority gender identity is likely to receive abuse targeted surrounding their identity, and abuse targeted at these individuals is likely to follow different dynamics.

Some data were collected retrospectively from Twitter’s archive search, which means abusive tweets removed through Twitter’s moderation efforts or deleted by users prior to collection will not have been captured. The levels of abuse we present, therefore, may be underestimates of the actual abuse experienced by public figures. As Twitter’s moderation efforts may be more focused on the most severe forms of abuse or on the most prominent public figures, our dataset is potentially biased towards less severe abuse and less prominent individuals.

This study was conducted sequentially, with data collection, annotation, and modelling occurring at different times for different domain-demographic groups. Data annotation uses the same schema across all groups, but, as discussed, annotation is done by 2 different groups (the expert annotator group remained the same), which may introduce uncertainty. The first model trained in this study utilised active learning, an effective but resource-intensive approach which could not be replicated for later models.

The variable time windows in data collection in this study represent variation between the groups studied. Arguably the real world events that occur within these time windows skew results, but one would struggle to be able to measure a “baseline” level of abuse through real world data collection. We take measures to account for variable times windows at multiple points in the analysis.

**Figure 8:**
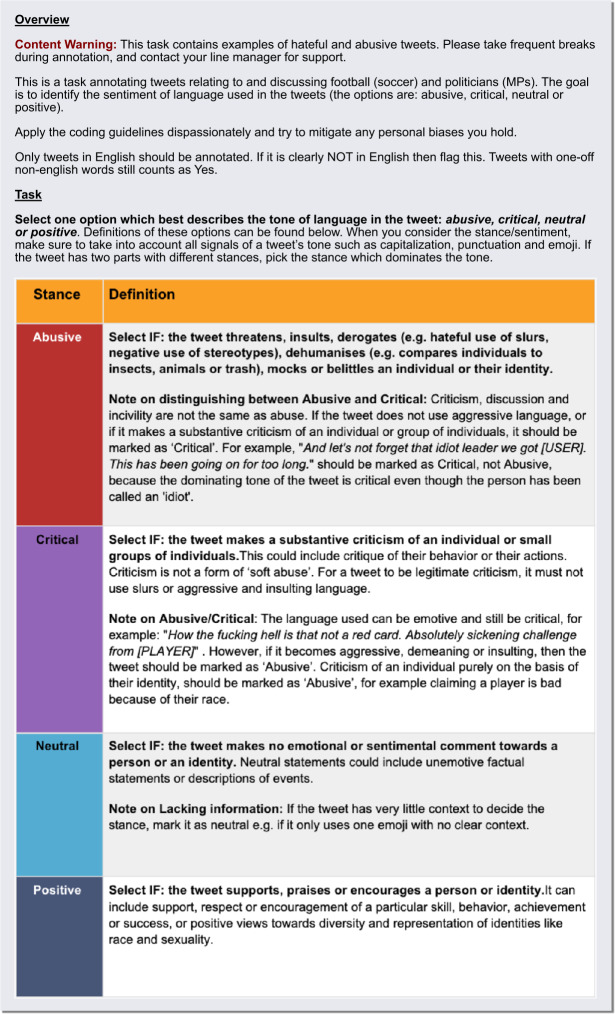
Instructions given to annotators

**Figure 9:**
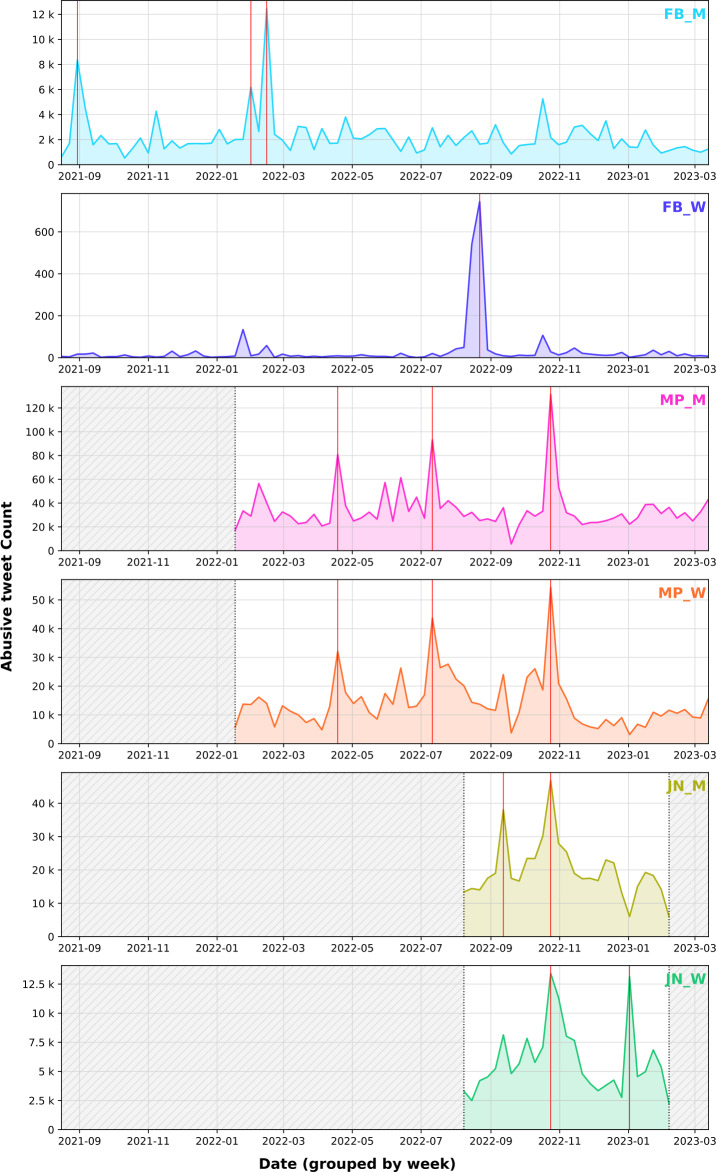
Abusive tweet counts by week for each group of public figures

We designed the annotation schema (Fig. 8) to differentiate between criticism and abuse. However, much of the abuse directed at public figures could indeed be interpreted as overly-profane or toxic forms of criticism. While annotators were given a strict schema to follow, we note that levels of annotator disagreement were higher in cases where the final majority label was either “critical” or “abusive”, highlighting the inherent ambiguity and nuanced distinction between these two categories. This poses a significant methodological challenge in this area of research, and may mean that some tweets classified as abusive in our research may be labelled as toxic criticism in other studies. As a result, our research may somewhat overestimate the levels of abuse compared to analyses using broader toxicity measures.

The models used to classify tweets do not match state of the art models in this task, achieving F1 scores between 0.61 and 0.68. This introduces error that may affect the findings presented. This is not a modelling paper, and we do not compare other models, so we cannot say whether this is a limitation of these models specifically or more a factor of the difficulty of this specific dataset. While there is some inconsistency between the modelling approaches that may be a limitation to this claim, the models do all use the same data collection and annotation procedures, and are derived from the same pre-trained model, and this approach does offer a more aligned way of comparing abuse across groups than e.g. using a set of models constructed through differing methodologies.

### Future work

As discussed, expanding beyond binary gender the would be a logical extension to this work. In addition to this, further work could be done to expand beyond a single demographic (gender) to better understand the dynamics of abuse across a range of identities.

Content analysis (with more nuance than whether content is abusive or not according to a machine learning model) of tweets targeted at public figures would provide a greater understanding of the themes contained within abuse, and could be combined with incorporation of data around real world events to provide more granular explanations of specific peaks in abuse.

Further research questions building on this work include developing a better understanding of the perpetrators of abuse, and how the affiliations and beliefs of perpetrators of abuse varies between groups of public figures. Extending beyond binary abuse classification to be able to measure the severity of abuse, and discern different forms of abuse (e.g. misogyny, racism), would open up avenues to explore the types of abuse received by public figures from different domains and demographics.

## Data Availability

The dataset of tweets analysed during the current study is not publicly available due to restrictions on sharing data collected from the Twitter API, as outlined in the API terms and conditions. We make available anonymised aggregate statistics, available from the corresponding author on reasonable request.

## Appendix A: Data collection

### A.1 Sourcing public figures

For footballers, we focus on the top UK leagues of the men’s and women’s game, namely the Men’s Premier League, consisting of 20 teams at any given point, and the Women’s Super League, with 12 teams at any given point. The exact number of players in each league is fuzzy, but we estimate the total number of eligible individuals to be around 1000 male footballers and 300 female footballers. Within politics, we focus on UK Members of Parliament (MPs), the most prominent public figures in UK politics as elected officials voted for by the public, constituting 650 individuals. Unlike footballers and MPs, journalists are not a finite group of individuals, adding complexity to the selection of individuals and data collection. As such, we use a list of UK journalists on Twitter [68] (now no longer maintained), selecting the top 3000 journalists in terms of Twitter follower numbers. We chose a larger number of journalists than MPs or footballers to account for the fact that there is no finite number, and to capture a range of abuse, given that abuse is not a phenomenon limited to the most popular journalists.

### A.2 Collecting public figure information

We scrape the official websites of the Premier League [69] and Super League [70], alongside the websites of individual clubs, to gather lists of eligible players. This information includes complete information on name, club, and nationality, and incomplete information on position, date of birth, height, and Twitter account (gender is implicit in the data collection process). For MPs, we collate a complete list using several sources [71] [72] [73] (the latter is no longer available). These provide complete information on name, gender, party, constituency, and incomplete information on Twitter account. The list of journalists [68] provides complete information on name, publisher, publication, job role, and Twitter account, and no information on gender.

We estimate gender for journalists using a hybrid approach, first obtaining a gender and probability from the first names of the 3000 journalists studied using genderize [74] (based on census data). In cases where this approach returned a gender with a probability less than 100% (912 entries, 30%), we prompt GPT-4 [75] with the full name and publication of the journalist, asking to indicate if it is aware of the journalist and what their gender is. In cases where the two approaches disagreed or GPT-4 did not indicate awareness of the individual (313 entries, 10%), we manually labelled gender using available resources online.

### A.3 Social media presence

Where records of Twitter profiles were incomplete (footballers and MPs), we use a combination of the Twitter API and desk research to assign Twitter profiles where they exist (some MPs and footballers are not present on Twitter). During data collection, changes in user name or deletions of account were recorded, as were any changes in affiliation (e.g. footballers transferring to other clubs, MPs losing their seat). Numbers of accounts and any analysis presented is inclusive of all accounts used for data collection through the entire data collection process.

## Appendix B: Data annotation

We used two different sets of annotators across the two domains, as we annotated the sets sequentially. Initial annotation rounds revealed high rates of annotator disagreement, with a large number of entries requiring expert annotation as a result. We use the same label schema for all domain and demographic pairs but use specific example tweets in the guidelines. We only employ annotators who pass a test of gold questions. Annotators were informed prior to accepting the task that the data would be used to train machine learning models as part of a research paper.

We employed 3375 crowdworkers for male footballers and 3513 for female footballers. The final number of crowdworkers was determined by how many were required to gather the required number of responses per input, taking into account failed gold-standard and attention check questions interspersed throughout annotation work by the platform. Crowdworkers were paid $0.20 per annotation, earning $11.30/hour on average. Each entry was annotated by 3 crowdworkers, with an additional two annotations required if no majority agreement ($\frac{2}{3}$) was reached, then sent for expert annotation if still no majority agreement ($\frac{3}{5}$) was reached. The average annotator agreement per entry was 68%, and the Cohen’s kappa was 0.50.

The demographic information available on these workers is limited due to use of a crowdsourcing platform. For male footballers, the annotators were from 41 countries, and from 48 countries for female footballers. The annotators for both datasets were primarily from Venezuela (56% and 64% respectively) and the United States (29% and 18% respectively).

For the MP and Journalist datasets, we employed 23 high-quality annotators from a Trust & Safety organisation. Annotators were paid $0.33 per annotation, earning $16.80/hour on average. Each entry received 3 annotations, then sent for expert annotation if no majority agreement was reached ($\frac{2}{3}$). For MPs, the average entry-wise agreement was 82% and the Cohen’s kappa was 0.67, compared to 85% and 0.66 for Journalists.

The annotators were screened from an initial pool of 36 annotators who took a test consisting of 36 difficult gold-standard questions (containing examples of all four class labels). The annotators had constant access to both a core team member from the service provider and from the core research team. Of the 23, 15 annotators self-identified as women, and 8 as men. The annotators were sent an optional survey to provide further information on their demographics. Out of 23 annotators, 21 responded to the survey. By age, 12 annotators were between 18-29 years old, 8 were between 30-39 years old, and 1 was over 50 years old. In terms of completed education level, 3 annotators had high school degrees, 8 annotators had undergraduate degrees, 6 annotators had postgraduate taught degrees, and 4 annotators had postgraduate research degrees. The majority of annotators were British (17), and other nationalities included Indian, Swedish, and United States. 12 annotators identified as White, with 1 identifying as White Other and 1 identifying as White Arab. Other ethnicities included Black Caribbean (1), Indian (1), Indian British Asian (1), and Jewish (1). Most annotators identified as heterosexual (14), with other annotators identifying as bisexual (3), gay (1), and pansexual (1). 2 chose not to disclose their sexuality. The majority stated that English was their native language (16), and 4 stated they were not native but fluent in the language. 1 chose not to disclose whether they were native English speakers or not. The majority of annotators disclosed that they spend 1-2 hours per day on social media (12). 4 annotators stated that they spent, on average, less than 1 hour on social media per day (but more than 10 minutes), and 5 stated they spend more than 2 hours per day on social media. Some of the annotators reported having themselves been targeted by online abuse (9), with 11 reporting ‘never’ and one preferring not to say.

An example of instructions given to annotators is displayed in Fig. 8. Due to the potentially harmful nature of the task, annotators were encouraged to regularly take breaks, and to contact their line manager or corresponding point of contact in event of any problems or concerns. Annotator pay was above US minimum hourly wage at the time on average for all jobs.

## Appendix C: Model performance

**Table 10:** Performance for candidate abuse classification models, with best performing model for each group in bold

Group	Description	Train size	F1 score	Precision	Recall
FB;M	Model trained on only FB;M data	3K	0.57	0.55	0.60
	Model trained on FB;W and FB;M data	6K	0.53	0.53	0.52
	Ensemble of two models: one trained on FB;M data, one on FB;W data	2*3K	0.57	0.67	0.50
	**Model trained using active learning (only on FB;M data)** [10]	**9.5K**	**0.65**	**0.65**	**0.64**
FB;W	Model trained on only FB;W data	3K	0.44	0.54	0.36
	Model trained on FB;W and FB;M data	6K	0.56	0.52	0.60
	**Ensemble of two models: one trained on FB;M data, one on FB;W data**	**2*3K**	**0.62**	**0.79**	**0.51**
MP;M	Model trained on only MP;M data	3K	0.66	0.62	0.70
	Model trained on MP;W and MP;M data	6K	0.67	0.66	0.67
	**Ensemble of two models: one trained on MP;M data, one on MP;W data**	**2*3K**	**0.68**	**0.71**	**0.65**
MP;W	Model trained on only MP;W data	3K	0.59	0.66	0.54
	Model trained on MP;W and MP;M data	6K	0.61	0.61	0.61
	**Ensemble of two models: one trained on MP;M data, one on MP;W data**	**2*3K**	**0.61**	**0.67**	**0.56**
JN;M	**Model trained on 3000 mixed JN tweets**	**2*3K**	**0.62**	**0.61**	**0.64**
JN;W	**Model trained on 3000 mixed JN tweets**	**2*3K**	**0.66**	**0.66**	**0.66**

## Footnotes

1 We note that 216 potential outliers were observed for Model 4. After carefully inspecting these data points and concluding they were genuine (and not as a result of data errors) we decided against excluding them from our analysis.

## Abbreviations

UK: United Kingdom
MPs: Members of Parliament
AC: audience contact
API: application programming interface
IRR: incident rate ratio
VIF: variance inflation factors
CD: Cook’s distance
